# Research on the factors that influence patients with colorectal cancer participating in the prevention and control of surgical site infection: Based on the extended theory of planned behaviour

**DOI:** 10.1111/hex.13355

**Published:** 2021-09-12

**Authors:** Lili Yao, Mingzhao Xiao, Yetao Luo, Lupei Yan, Qinghua Zhao, Yuerong Li

**Affiliations:** ^1^ Department of Anesthesiology The First Affiliated Hospital of Chongqing Medical University Chongqing China; ^2^ Department of Urology The First Affiliated Hospital of Chongqing Medical University Chongqing China; ^3^ Department of Nosocomial Infection Control The Second affiliated Hospital of Army Medical University Chongqing China; ^4^ Department of Nursing The First Affiliated Hospital of Chongqing Medical University Chongqing China

**Keywords:** colorectal cancer, health and safety, infection control, patient participation

## Abstract

**Background:**

The most common and severe type of nosocomial infection in patients with colorectal cancer is surgical site infection (SSI). Patient‐related factors are an important components of SSI. So it is necessary to participate in SSI prevention and control. It is important to identify the factors that influence patients' participation behaviour and to explore the mechanism of these effects.

**Methods:**

A total of 580 patients with colorectal cancer completed relevant measures. Based on the extended theory of planned behaviour, a structural equation model was used to analyse the relationship among the influencing factors.

**Results:**

The factors influencing participation of patients with colorectal cancer in SSI prevention and control were participation intention, participation ability, self‐efficacy, participation attitude, perceived medical staff support, trust in physicians and social support. The direct effect coefficients of participation intention, participation ability and physician trust on SSI prevention and control behaviour were 0.67, 0.21 and 0.11, respectively. Self‐efficacy, participation attitude, perceived medical staff support and social support indirectly affect participation behaviour through participation intention, and their effect values are 0.21, 0.11, 0.11 and 0.08, respectively.

**Conclusions:**

Based on the structural equation model developed in this study, targeted intervention measures should be implemented to mobilize the intention and enthusiasm of patients with colorectal cancer to participate in the prevention and control of SSI.

**Patient or Public Contribution:**

Patients or public contribute to spreading research findings, and promote broad participation in the implementation of policies or strategies.

## 1 BACKGROUND

Colorectal cancer (CRC) is a malignant tumour occurring in the colon or rectum, and it is the most common type of malignant tumour in the digestive tract. The incidence and mortality of CRC are still on the rise. According to the cancer statistical report of 2018, the incidence and mortality of CRC ranked third and fifth among malignant tumours in China, respectively, with 376,000 new cases and 191,000 deaths a year.^1^ CRC is usually treated by surgical resection supplemented by radiotherapy and chemotherapy. The incidence of surgical site infection (SSI) is approximately 20%^2^, ^3^; the incidence of SSI is significantly higher in low‐ and middle‐income countries than in high‐income countries.^4^ SSI is one of the most common health‐related infections, but its importance as a global health priority is not fully understood.^5^ CRC patients with SSI usually result in prolonged hospital stay, heavy expenditure, poorer life quality, higher mortality and lower long‐term survival rate. A study showed that the risk of death in patients with SSI is 2–11 times higher than that of patients without SSI, and the annual cost of health care‐related economic expenditure is approximately $3.5 billion to 10 billion. Because of the time lag of returning to work, it will lead to indirect economic losses due to missing work or unemployment.^6^

SSI is one of the most common health care‐related infections worldwide and poses a heavy burden on health care systems and individual patients. Various risk factors for SSI have been identified, which can be divided into patient‐and procedure‐related risk factors and other risk factors. Among them, patient factors account for a large proportion.^7^, ^8^ Yokoe et al.^9^ found that up to 60% of SSIs can be avoided through evidence‐based interventions, and patients play an important role in these interventions. Wang et al.^10^ also found that patient participation could significantly reduce the incidence of SSI and improve satisfaction. From an international point of view, the World Health Organization posited that ‘patients have the right and responsibility to participate in their own health care’ at the primary health care conference in 1978 and proposed the Patient for Patient Safety action plan in 2004 to encourage patients to participate in the improvement of care quality and safety. Patient participation is considered to be one of the main factors in improving quality and safety and is considered to be an effective intervention to promote safe care.^11^

Patient is the center of medical practice. Ensuring the safety of patients is the bottom line to improve medical quality. Encouraging patients to participate in patient safety is the focus of continuous efforts in various countries and medical systems, and patients' participation in nosocomial prevention and control is the initial core content in patient safety. Participation is a vague concept in medical practice, and patient participation can be defined as a concept that involves three scientific concepts of care: learning, caring relationship and reciprocity (defined attributes).^12^ At present, as the basic rights of patients and an important measure to ensure the safety of patients, patient participation has received increasing attention in the field of health. Increasing attention has been paid to patients' participation in the prevention and control of nosocomial infections, especially the high incidence of SSI, but there is a relative lack of research on the influencing factors and mechanism of patients' participation in the prevention and control of SSI.

The theory of planned behaviour (TPB) was developed by Ajzen on the basis of the rational behaviour theory. This theory holds that individual behaviour intention is the best predictor of behaviour and has a direct influence on behaviour; besides, intention is influenced by behaviour attitude, subjective norms and perceptual behaviour control. All of these have an indirect effect on behaviour through intention. In particular, perceptual behaviour control can also act directly on behaviour. In this study, support from medical personnel, relatives and friends reflects subjective norms. Perceptual behaviour control reflects patients' perception of factors that promote or hinder participation in behaviour, including their own knowledge, ability and health status. Self‐efficacy and participation ability belong to this category. Wu^13^ found that trust between medical staff and patients can affect patient participation, thus adding to the theoretical model.

Currently, perioperative nursing care has changed from a task‐oriented, problem‐centred discipline to a patient‐centred professional discipline. This change involves the research and implementation of evidence‐based practice to improve the quality of patient care. There is still a lack of literature that directly uses the TPB to describe and explain perioperative practice behaviour. Medical staff can use this theory to understand the intention behind the behaviour of surgical patients to ensure the safety of care and optimize clinical outcomes.^14^ Therefore, this study is based on the extended TPB to establish a mechanism model for influencing factors of CRC patients' participation in the prevention and control of SSI to provide a reference basis for medical institutions and related departments to formulate strategies for SSI.

## 2 METHODS

### 2.1 Study design, setting and participants

The current study used a cross‐sectional design and a questionnaire survey. The inclusion criteria were as follows: Colonoscopy or pathological examination that finally led to the diagnosis of colon cancer or rectal cancer, and elective laparoscopic surgical treatment; age over 18 years; without mental disorder; stable physical status; and provision of agreement to participate in this study. The exclusion criteria were as follows: Patients who underwent laparotomy or robot‐assisted surgery, or withdrew from the study due to unexpected reasons.

Structural equation modelling was used as the main statistical analysis method. A structural equation model is suitable for analysing large samples. If the sample size is analysed by the number of observed variables in the model, the ratio of the sample size to the observed variables should be at least 20:1.^15^ This study includes 21 observed variables, calculated 20 times, and the required sample size is 420. Taking into account a potential 20% attrition rate, the final sample size is determined to be 525.

### 2.2 Measures

In addition to the general information questionnaire, the following measures were used in this study: The promoting patient participation scale, the intention and behaviour measures of CRC patients participating in the prevention and control of SSI, the Chinese patient participation ability scale, the patient participation attitude questionnaire, the Wake Forest physician trust scale, the general self‐efficacy scale and the perceived social support scale.^13^, ^16^, ^17^, ^18^, ^19^, ^20^, ^21^ These measures have been shown to have good reliability and validity.

### 2.3 Data collection

From May 2020 to October 2020, data were collected in the gastrointestinal surgery ward in a Tertiary Hospital affiliated with the university in Chongqing, China. Before the investigation, the researchers contacted the head of the investigation department, explained the purpose and significance of the research, obtained the ethical approval certificate and obtained support and assistance. During the survey, the researchers explained to the patients how to fill in the study objectives, emphasized the anonymous nature of the study and collected data for scientific purposes only. After the questionnaires were completed, the researchers checked the completeness of the answers and collected the questionnaires.

### 2.4 Statistical analysis

The statistical software package IBM SPSS Statistics 23.0 (IBM Corp.) was used for data entry and analysis. The demographic data of the patients were described by frequency and percentage. The Pearson correlation coefficient was used to estimate the correlation between variables. Confirmatory factor analysis (CFA) was used to test the relationship between latent variables and observed variables of the measurement model. We used IBM SPSS Amos 24.0 software to build the model, used the maximum likelihood estimation method to estimate the model parameters and optimized the model according to the revised index. *p* < .05 was considered statistically significant.

## 3 RESULTS

### 3.1 Demographic characteristics

In this study, a total of 608 questionnaires were distributed, and 580 valid questionnaires were collected, with an effective response rate of 95.39%. Among the respondents, there were 346 males (59.66%) and 234 females (40.34%). A total of 125 patients (21.55%) were over 71 years old; 44.83% had an abnormal body mass index and 54.31% had complications. Only 47 (8.10%) CRC patients accepted preoperative neoadjuvant therapy, including radiotherapy, chemotherapy and radio‐ and chemotherapy in combination, as shown in Table 1.

**Table 1:** Sociodemographic characteristics (*N *= 580)

Variables		*N*	Percent (%)
Gender			
Male		346	59.66
Female		234	40.34
Age (years)			
18–30		8	1.38
31–50		130	22.41
51–70		317	54.66
≥71		125	21.55
BMI (kg/m^2^)			
<18.5		62	10.69
18.5–23.9		320	55.17
24–27.9		169	29.14
≥28		29	5.00
Occupation			
Civil servant		25	4.31
Business manager		34	5.86
Worker		70	12.07
Farmer		141	24.31
Freelancer		40	6.90
Self‐employed person		24	4.14
Retired personnel		160	27.58
Other		86	14.83
Marital status			
Unmarried		4	0.69
Married		524	90.34
Widowed		28	4.83
Divorce		24	4.14
Education level			
Elementary school and below		163	28.10
Junior high school education		199	34.31
High school or technical secondary school degree		114	19.66
College degree		54	9.31
Bachelor's degree and above		50	8.62
Residence			
City		347	59.83
Township		134	23.10
Rural area		99	17.07
Medical burden			
Completely unburdened		7	1.21
Basically no burden		115	19.83
Have a certain burden		258	44.48
Heavy burden		200	34.48
Length of hospital stay before surgery (days)			
<3		130	22.42
3–7		229	39.48
>7		221	38.10
Complications			
No		265	45.69
Yes		315	54.31
Preoperative neoadjuvant therapy			
No		533	91.90
Yes		47	8.10

Abbreviation: BMI, body mass index.

### 3.2 Descriptive analysis of the influencing factors of CRC patients participating in the prevention and control of SSI

The intention, behaviour, trust, attitude, self‐efficacy, perceived medical staff support, social support and participation ability scores were 110.32 ± 12.69, 94.32 ± 15.59, 36.50 ± 4.61, 21.34 ± 4.51, 25.99 ± 5.66, 30.64 ± 3.96, 63.14 ± 10.15 and 65.57 ± 10.1, respectively (Table 2).

**Table 2:** Descriptive analysis of influencing factors for patients with colorectal cancer to participate in the prevention and control of surgical site infections

Variable	Mean	Standard deviation	Skewness	Kurtosis
Physician trust	36.50	4.61		
Charity	19.23	2.95	0.03	−0.07
Technical competence	17.27	2.12	0.04	−0.05
Participation attitude	21.34	4.51		
Medical support	30.64	3.96		
Social support	63.14	10.15		
Intrafamily support	22.47	3.31	−0.03	0.01
Out‐of‐family support	40.66	7.41	−0.09	0.02
Participation ability	65.57	10.10		
Information acquisition	11.28	2.04	−0.02	−0.08
Independent decision	9.42	3.52	0.05	−0.04
Communication	19.26	3.37	−0.02	−0.06
Emotional management	25.61	3.04	−0.05	−0.36
Self‐efficacy	25.99	5.66		
Intention	110.32	12.69		
Appeal participation	29.25	4.11	−0.04	−0.05
Decision participation	31.35	5.52	−0.18	−0.06
Inquiry supervision	16.70	2.59	−0.07	−0.23
Information interaction	14.04	1.69	−0.08	0.05
Caring participation	18.98	1.56	−0.05	0.02
Behaviour	94.32	15.59		
Appeal participation	24.60	4.58	0.19	−0.07
Decision participation	26.33	5.94	0.11	−0.06
Inquiry supervision	14.08	2.69	0.03	−0.04
Information interaction	12.99	2.28	0.01	−0.18
Caring participation	16.30	2.57	0.08	0.06

### 3.3 Correlation coefficients among variables

Physician trust, participation attitude, perceived medical staff support, perceived social support, participation ability and self‐efficacy were positively correlated with the intention and behaviour of infection prevention and control at the surgical site, as detailed in Table 3.

**Table 3:** Correlation coefficient between variables in patients with colorectal cancer (*N *= 580)

Variable	Physician trust	Participation attitude	Medical support	Social support	Participation ability	Self‐ efficacy	Intention
Physician trust	1						
Participation attitude	0.525*	1					
Medical support	0.027	0.139*	1				
Social support	0.384*	0.391*	0.074	1			
Participation ability	0.52*	0.65*	0.205*	0.395*	1		
Self‐efficacy	0.431*	0.493*	0.051	0.342*	0.651*	1	
Intention	0.297*	0.367*	0.208*	0.282*	0.393*	0.421*	1
Behaviour	0.409*	0.491*	0.222*	0.392*	0.52*	0.495*	0.73*

* *p* < .001.

### 3.4 Structural equation modelling of the factors affecting CRC patients' participation in the prevention and control of SSI

#### 3.4.1 Confirmatory factor analysis

Table 4 shows the CFA test of each structure of the model. All standardized factor load estimates were over 0.4 (*p* < .05). The analysis indicated that the data are suitable for modelling.

**Table 4:** Confirmatory factor analysis

		Factor loading	*p*	IFI	CFI	RMSEA	McDonald's *ω*
Participation attitude							
Total							0.868
Medical support							
Total							0.867
Self‐efficacy							
Total							0.866
Physician trust				0.910	0.909	0.079	0.804
Charity		0.861	<.001				
Technical competence		0.752	<.001				
Social support				0.905	0.905	0.079	0.902
Intrafamily		0.867	<.001				
Out‐of‐family		0.876	<.001				
Participation ability				0.957	0.957	0.068	0.912
Information acquisition		0.703	<.001				
Independent decision		0.728	<.001				
Communication		0.870	<.001				
Emotional management		0.803	<.001				
Intention				0.948	0.948	0.072	0.946
Appeal participation		0.801	<.001				
Decision participation		0.913	<.001				
Inquiry supervision		0.747	<.001				
Information interaction		0.433	<.001				
Caring participation		0.619	<.001				
Behaviour				0.951	0.951	0.075	0.939
Appeal participation		0.942	<.001				
Decision participation		0.887	<.001				
Inquiry supervision		0.869	<.001				
Information interaction		0.469	<.001				
Caring participation		0.750	<.001				

Abbreviations: CF, comparative fit index; IFI, incremental fit index; RMSEA, root mean squared error of approximation.

#### 3.4.2 Initial model assumption

**Figure 1:**
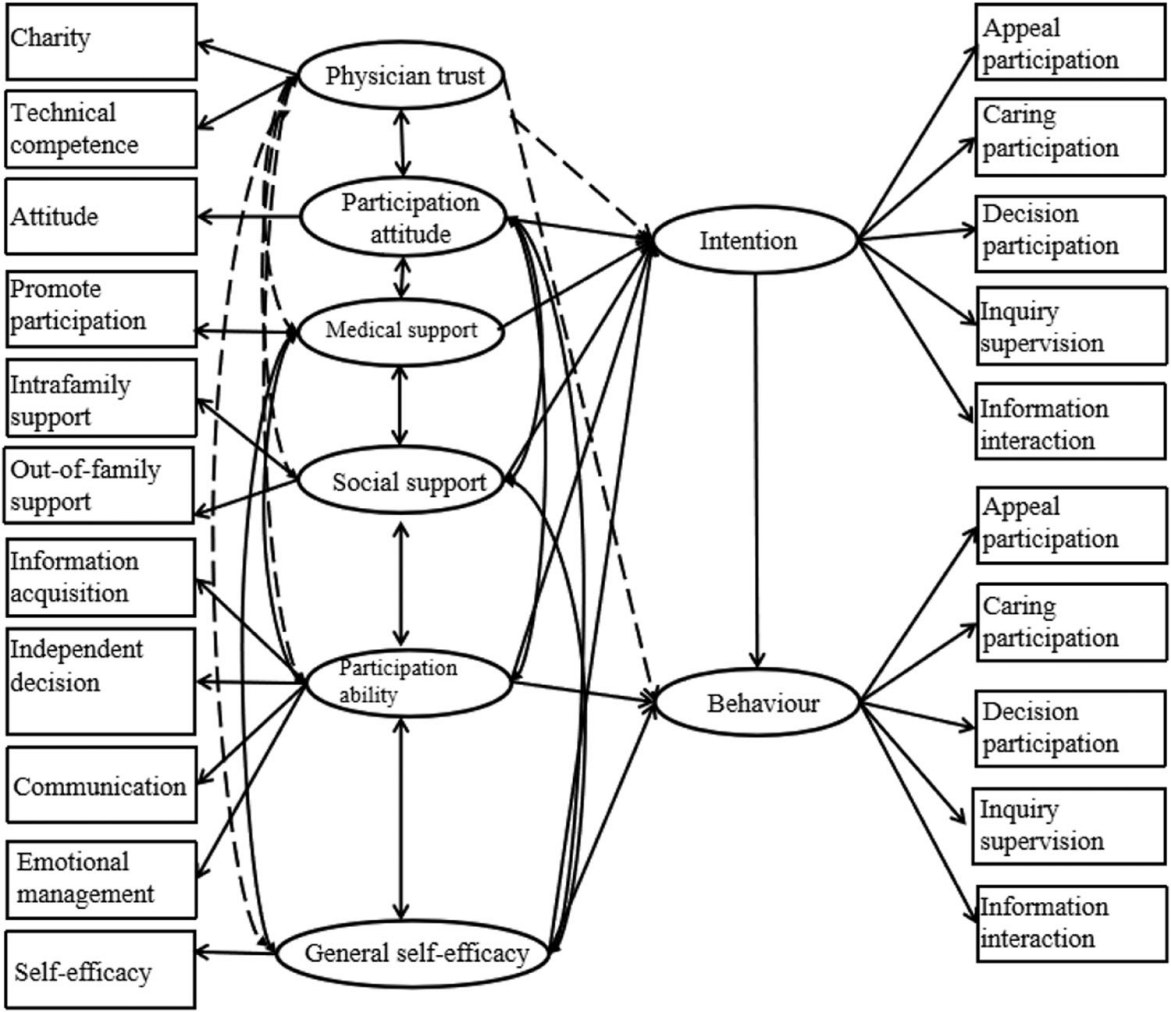
Initial model assumptions

In the model, we established the following hypothesis based on an extended TPB: Participation ability and general self‐efficacy directly affect participation behaviour, participation attitude and perceived medical staff support and social support affects participation behaviour through participation intention, as shown by the solid line in Figure 1. Physician trust can affect patient participation, which is shown by the dotted line in Figure 1. The general self‐efficacy scale, the promoting patient participation scale and the patient participation attitude questionnaire measures were all unidimensional and included only one observed variable, without a latent variable, so we replaced the latent variable with the observed variable; see Figure 1 (ovals, rectangles).

#### 3.4.3 Model modification

**Figure 2:**
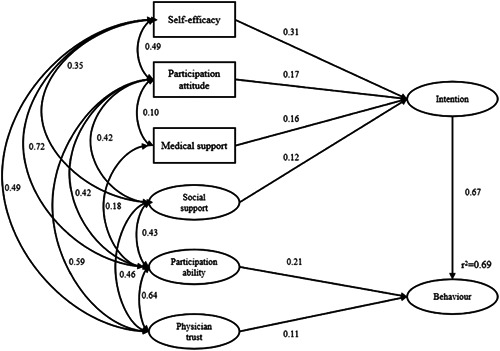
Final model path and standardized regression coefficients

Before constructing the initial model, according to the results of the correlation coefficient, the relevant variables with *p* > .05 were deleted (i.e., perceived medical staff support and physician trust, perceived medical staff support and perceived social support, perceived medical staff support and self‐efficacy). After constructing the initial model, three paths with *p* > .05 were deleted: ‘physician trust → intention’ (*β* = .010, *p *= .878), ‘participation ability → intention’ (*β* = .063, *p *= .474), and ‘self‐efficacy → behaviour’ (*β* = .026, *p *= .523). In the final model, the total effect values of self‐efficacy, participation attitude, perceived medical staff support, social support, participation ability, physician trust and intention on behaviour were 0.21, 0.11, 0.11, 0.08, 0.21, 0.11 and 0.67, respectively. These variables explained 69.00% of the variation in behaviour. The modification of the index is based on Table 5.^15^ The final model is shown in Figure 2. The *p* value was less than .05 for all path coefficients.

**Table 5:** Modification basis for model indices

Index	*χ* ^2^	*χ* ^2^/*df*	GFI	PGFI	NFI	IFI	CFI	RMSEA
Initial model	1257.328	7.396	0.861	0.609	0.843	0.862	0.861	0.105
Revised model	724.459	4.509	0.901	0.623	0.91	0.929	0.928	0.078
Reference model		1–5	>0.90	>0.50	>0.90	>0.90	>0.90	<0.08

Abbreviations: *df*, degrees of freedom; GFI, goodness‐of‐fit index; NF, normed fit index; PGFI, parsimony goodness of fit index.

## 4 DISCUSSION

### 4.1 CRC patients have the intention to participate in the prevention and control of SSI, but participation behaviour is inferior to the intention

For surgical patients, SSI of postoperative complications has always been the focus of medical attention. This study focuses on CRC patients with a high incidence of SSI. Through investigation, it was found that CRC patients intentionally participated in the prevention and control of partial infection after the operation, with a score of 110.32 ± 12.69. It is suggested that CRC patients have a high level of intention to participate in the prevention and control of SSI, and there is a need to participate in such behaviour. Levinson et al.^22^ also found that almost all respondents (96%) had a strong behavioural intention with respect to disease diagnosis and treatment and wanted to communicate with health care providers. Although CRC patients have a strong intention to participate in the prevention and control of SSI, the actual behaviour is less common, with a score of 94.32 ± 15.59, which is lower than that of intention. The scores of all dimensions involved in surgical infection prevention and control are also lower than those of intention, which is consistent with the conclusions of Keating et al.^23^

The behaviour of CRC patients participating in the prevention and control of SSI varies according to the content. The order of participation behaviour score is as follows: decision participation (26.33 ± 5.94) > appeal participation (24.60 ± 4.58) > caring participation (16.30 ± 2.57) > inquiry supervision (14.08 ± 2.69) > information interaction (12.99 ± 2.28). Female, well‐educated and healthy individuals were more likely to play an active role in decision participation.^22^ The previous research^24^ found that, because of the gradient of authority and patients' concern that the appeal will affect the harmonious relationship between medical staff and patients, the score of appeal participation is generally low and the scores of information interaction and caring participation are rather high; however, we obtained different results. With the concept of ‘patient‐centred care’ deeply rooted in the hearts of the people and due to the renewed focus on patients' awareness of rights, the behaviour of CRC patients participating in SSI prevention and control is gradually becoming different from patient behaviour in ordinary health care.

### 4.2 Participation behaviour is affected by many factors

#### 4.2.1 Analysis of the influence of intention on behaviour

The intention of CRC patients to participate in the prevention and control of SSI refers to the behavioural motivation and ideological tendency before participating in the behaviour of SSI prevention and control. Intention is the most important behaviour predictor in the planned behaviour theory. In this study, the correlation coefficient between CRC patients' intention to participate in SSI prevention and control and prevention and control behaviour was .73 (*p* < .001). In the structural equation model constructed in this study, the effect of intention on behaviour is the largest, 0.67, which is consistent with the conclusion of Hagger's research.^25^ According to the model, self‐efficacy, participation attitude, medical support and social support have a direct effect on CRC patients' intention to participate in SSI prevention and control, and the effect coefficients were .31, .17, .16 and .12, respectively. This result suggests that measures can be taken to improve the self‐efficacy and participation attitude of CRC patients, to strengthen medical support and social support, to directly improve participation intention and indirectly promote their participation in SSI prevention and control.

#### 4.2.2 Analysis of the influence of participation attitude on behaviour

This study found that participation attitude indirectly positively affects participation behaviour through participation intention, and the total effect value on behaviour is 0.11. Patient participation in the prevention and control of SSI has changed the role of patients from the traditional ‘receiver’ to the current role of ‘collaborator’. Patients' attitudes and cognition towards this new role have a significant impact on their intention to participate in prevention and control and their actual participation behaviour. Davis et al.^26^ found that 1 in 10 hospitalized patients suffered an adverse event as a result of treatment or surgery. If patients can consciously and actively participate in the diagnosis, treatment and nursing activities, the risk of adverse events will be reduced. However, such a positive attitude does not directly affect the participation behaviour, but plays a role through the participation intention. It should be a priority for medical institutions to mobilize the attitude of patients to participate, strengthen their participation intention and optimize their participation behaviour.

#### 4.2.3 Analysis of the influence of medical support on behaviour

Medical staff who promote patient participation and patient perception to support their participation in medical care is an important embodiment of ‘patient‐centred’ practice. In this study, the score of the perceived medical support of CRC patients was 30.64 ± 3.96. Through participation intention and indirect positive prediction of participation behaviour, the total effect value was 0.11. A qualitative study shows that CRC patients expect the support of health care staff during hospitalisation.^27^ Most medical staff support patients' participation in medical safety and are willing to actively communicate with patients on related issues. Patient participation can enable medical staff to fully understand patient information, clarify safety issues, help to meet the needs of patients and achieve better results.

#### 4.2.4 Analysis of the influence of physician trust on behaviour

The results of this study showed that the correlation coefficient between CRC patients' trust in physicians and their participation in SSI prevention and control behaviour was 0.409 (*p* < .001), which was moderately positively correlated. The trust of CRC patients in physicians can directly predict their participation in SSI prevention and control behaviour (the path coefficient is 0.11). Trust is an important influencing factor of patient participation, patient satisfaction and physician–patient relationships. If a mutual trust relationship established between CRC patients and medical staff during perioperative stage, they may develop good compliance of SSI prevention and control behaviour, and better participation in many areas of health care. Trachtenberg et al.^28^ found that patients' trust in physicians promotes patients' participation in their own health care, and patients who trust health care staff are more likely to reach a consensus with them, thus reducing conflicts in health care decision‐making.

#### 4.2.5 Analysis of the influence of participation ability on behaviour

Patient participation ability includes self‐management ability and health literacy.^29^ Patient self‐management refers to patients' ability to choose treatment options and cope with psychological and physiological changes, as well as lifestyle changes, according to their own symptoms in daily life. The ability of self‐management is not limited to compliance with treatment; more importantly, the physical and psychological coping abilities of patients should be integrated into the long‐term process of dealing with diseases. Self‐management ability will contribute to recovering faster for CRC patients after surgery. Katz et al.^30^ found that patients with high health literacy showed stronger ability to collect information, and they asked health care workers more questions. Patients with low health literacy are less likely to ask questions because they are worried that health care workers will think that their ability to understand the disease and treatment information is limited. The current study found that participation ability plays a direct positive predictive role in participating in SSI prevention and control behaviour, and the total effect value is 0.21. How to improve the participation ability of CRC patients is a topic worth discussing.

#### 4.2.6 Analysis of the influence of social support on behaviour

In this study, it was found that the score of perceived social support in CRC patients was 63.14 ± 10.15, which was indirectly positively predicted by participation in SSI prevention and control intention. Patients undergoing surgery often have the need for love and belonging, especially the support and care of relatives and friends, because they are anxious and worried about postoperative recovery. Relatives and friends are an important part of the patient's social support system, and the attitude and behaviour of relatives and friends will affect the patient's participation behaviour. A previous study found that inpatients obtained the support and encouragement from families, which promoted their active participation and positive cooperation with treatment.^31^

#### 4.2.7 Analysis of the influence of self‐efficacy on behaviour

This study showed that the total effect value of self‐efficacy on the behaviour of infection prevention and control at the surgical site was 0.21, which indirectly affected the behaviour by affecting the intention of participation. The expression of self‐efficacy is the subjective judgement and evaluation of an individual's behavioural ability. Empowering surgical patients to improve self‐efficacy is a new concept in the health care field. This action can effectively improve medical quality. McAlearney et al.^32^ found that empowerment is a process through which patients are better able to weigh decisions and behaviours that affect their health.

### 4.3 Thoughts and suggestions on promoting rational participation in the prevention and control of SSI in CRC patients

This study found that CRC patients have a high intention to participate in the prevention and control of SSI, which is a good and noteworthy aspect. Medical institutions and medical staff should respect their intention to participate. A study of SSI‐related knowledge and awareness of prevention and control among 52 patients found that only 60% of patients had received a brochure on SSI knowledge in the hospital, and 16% of the respondents said that they had not received any training in related knowledge at all.^33^ Knowledge training and health education on the prevention and control of SSI during the perioperative period should be strengthened, enthusiasm for participation should be promoted and surgical patients should be empowered. Medical personnel should initiatively communicate with patients, encourage patients to express confusions, consider patients as the cooperative partner. Anderso et al.^34^ found that cooperative participation will be developed and accepted as a mainstream form of patient participation; therefore, medical staff should also actively pay attention to the desire of patients to participate in information interaction, meet the information needs of patients in a variety of ways and take the initiative to provide patients with disease‐ and treatment‐related information in the process of communication and interaction between medical staff and patients. Different communication skills should be utilized to optimize the communication process, to improve the quality of communication, to ensure that patients correctly understand the information provided on SSI and, with the help of mobile medical technology, to provide patients with continuous and comprehensive information to fully grasp the relevant knowledge.

## 5 CONCLUSION

In this study, structural equation modelling revealed that the total effect of participation intention was the largest among the influencing factors of CRC patients participating in SSI prevention and control behaviour, which directly affected participation behaviour. Participation ability and physician trust directly positively predict participation behaviour. Self‐efficacy, participation attitude, perceived medical staff support and social support indirectly positively predict participation behaviour by influencing the intention to participate in SSI prevention and control. It is suggested that to promote the implementation of CRC patients' participation in the prevention and control of SSI, it is necessary to maximize their willingness to participate, pay attention to the relationship between family and social networks and strengthen social support and medical staff support. CRC patients should be encouraged to develop a desire to participate in the prevention and control of SSI and strengthen their sense of self‐efficacy. Effective measures can be taken to intervene directly and improve the trust and communication between medical staff and patients. In addition, based on the model constructed in this study, controllable and effective policies can be implemented to ensure that CRC patients participate in the prevention and control of SSI.

## 6 LIMITATIONS

A limitation of this study is that we adopted convenience sampling instead of random sampling. The representativeness of the sample may be insufficient, and there are certain limitations in the extrapolation of results. In addition, due to limited time, resources and funding, only one medical institution was investigated. In future studies, the sample size should be expanded, and multicentre and large‐sample surveys should be conducted.

## CONFLICT OF INTERESTS

The authors declare that there are no conflict of interests.

## AUTHOR CONTRIBUTIONS

All authors have made substantial contributions to the work are as follows: Lili Yao contributed to study design and article drafting. Yetao Luo contributed to data analysis and data interpretation. Lili Yao and Lupei Yan contributed to data collection. Mingzhao Xiao, Qinghua Zhao and Yuerong Li contributed to design of the study and thoroughly revised the manuscript. All authors have read and approved the final manuscript.

## ETHICS STATEMENT

This study was approved by the Ethics Committee of the First Affiliated Hospital of Chongqing Medical University (No. 2019‐131). Informed consent was obtained from all participants.

## Data Availability

Data are available upon reasonable request. Data can be shared upon reasonable request.
